# Out-of-pocket payments and loss of income among long-term breast cancer survivors in Germany: a multi-regional population-based study

**DOI:** 10.1007/s11764-022-01293-x

**Published:** 2022-12-02

**Authors:** Jana Schneider, Diego Hernandez, CAESAR study group, Michael Schlander, Volker Arndt, on behalf of the CEASAR study group

**Affiliations:** 1 Medical Faculty Heidelberg, Heidelberg University, Heidelberg, Germany; 2 Division of Health Economics, German Cancer Research Center (DKFZ), Heidelberg, Germany; 3 Medical Faculty Mannheim, Heidelberg University, Mannheim, Germany; 4 Unit of Cancer Survivorship, Division of Clinical Epidemiology and Aging Research, German Cancer Research Center (DKFZ), Heidelberg, Germany

**Keywords:** Breast cancer, Financial burden, OOP payments, Income loss, Long-term survivors, Population-based study

## Abstract

**Purpose:**

This study aims to examine the magnitude of out of pocket (OOP) payments and income loss, as well as to identify socioeconomic and clinical factors among long-term breast cancer (BC) survivors in Germany.

**Methods:**

We examine data from 2654 long-term BC survivors in Germany that participated in the “CAncEr Survivorship – A multi-Regional population-based study” (CAESAR) and who were at least 5 years post diagnosis. BC-related OOP payments and income loss both within the 12 months prior to the survey were analyzed. Two-part regression models were performed to identify socioeconomic and clinical factors.

**Results:**

OOP payments were incurred by 51.9% of survivors with a total mean spending of 566 euros. Income loss was present among 9.6% of survivors and averaged 5463 euros among those reporting such. Socioeconomic and clinical factors associated with higher OOP payments (*p* ≤ 0.05) included age at time of diagnosis (65–79 years), education (10–11 years), (early) retirement, stage of diagnosis (stage III), time from diagnosis (more than 10 years), comorbidities (at least 1), and the use of rehabilitation services. Regarding income loss, age at time of diagnosis (50–59 years), (early) retirement, stage of diagnosis (stage II), time from diagnosis (5–7 years), comorbidities (at least 1), and receiving chemotherapy treatment were associated with higher losses.

**Conclusions:**

For some survivors in Germany, financial burden can be considerably high despite comprehensive healthcare and support from social security.

**Implications for Cancer Survivors:**

OOP payments related to domestic help and nursing staff as well as to outpatient care are most frequent.

## Introduction

Breast cancer (BC) is the most frequently occurring cancer type in women, with approximately 69,900 new cases in Germany in 2018 [1]. The age-standardized incidence rate—impacted by the introduction of mammography screening in 2005—increased by 10.6% from the decade 2003/2004 to 2013/2014 [2]. Nevertheless, a decline of the age-standardized BC mortality rate has been observed from 2003/2004 to 2017/2018 by 15.4% [2]. Due to improved survival rates and the demographic ageing, the number of cancer survivors is increasing [1, 3–8]. There have been 1.03 million BC survivors in Germany by end of 2017. More than 71% of these BC survivors live at least 5 years post BC diagnosis and can therefore be considered as long-term survivors [5]. While 50.3% of females were 65 years or older at BC diagnosis, 49.7% were aged between 15 and 64 years at BC diagnosis and thus affected at working ages [5].

Breast cancer represents a significant financial burden not only for the society but also for the patient [4, 5, 7–10]. Financial burden may be attributed to both direct and indirect costs. Direct costs are incurred from use of healthcare services, for which out of pocket (OOP) payments may apply. In addition, patients may incur non-medical costs in connection with the provision of medical services. Indirect costs may consist of loss of income due to reduced work productivity or opportunity [10–13]. Moreover, financial burden due to cancer is not limited to the initial treatment phase, as health impairments and recurrences, metastases, or secondary tumors cause long-term effects on healthcare needs and the ability to work [4, 5, 14–16]. Direct medical costs have been perceived to be significant beyond 1 year after the breast cancer diagnosis, while non-direct medical costs to be even larger in amount in the long-term than in the short-term [16]. Direct medical costs that are particularly important among long-term breast cancer survivors include those for treatment of side effects, prosthesis, and other equipment that help women feel comfortable after surgery [17–19]. Direct non-medical costs comprise in greater extent those from clinic visits (transportation, overnight stays), support and assistance (childcare, housekeeping, distraction, counseling, household improvements, restaurant meals), and in lesser text those from administration (telephone bill, insurance premium) and adjustment for weight changes (clothes, cosmetics) [17–19]. Indirect costs are as well persistent for breast cancer survivors in the long-term and have been noted to be at least as equally important as direct costs in the composition of financial burden [17, 19]. The majority of survivors report income losses as a result of either reduced working hours, discontinued work or early retirement [17, 19, 20].

In Germany, 88% of the population are covered by statutory health insurance (SHI), while 11% are covered by private insurance. Additionally, the social security system provides additional benefits [21–24]. Nevertheless, access to healthcare and social security benefits does not fully protect patients from either OOP payments or income loss [25, 26]. Patients may incur in OOP payments in several circumstances. Co-payments within the SHI are 10% of the reference price of the healthcare service used, limited to a maximum of 10 euros per service. In addition, a co-payment of 10 euros per day is required for each of the following: prescription medicine, nursing staff, and domestic help, as well as inpatient care. Co-payments for inpatient care are, however, limited to 28 days per year, equivalent to a total amount of 280 euros per year. Travel expenses for outpatient care are covered in accordance with the general co-payment regulations, but only in exceptional cases defined by the Federal Joint Committee (G-BA)—the highest decision-making body in the German healthcare system—. For instance, a co-payment applies in each round trip to every clinic visit for radio- and chemotherapy [22, 24]. In case of treatment relocation that requires longer travel distances, additional costs must be completely born by the patient [24]. Nevertheless, co-payments are required to be limited to 2% of the annual gross household income and 1% in cases of chronic illness. Among individuals receiving social benefits, co-payments are limited to an annual fixed fee of 98 euros and in individuals with chronic diseases, 49 euros [22, 27, 28]. Yet, Germany had the largest percentage increase in OOP payments among high-income countries, close to 87% from 2000 to 2010, and these costs accounted for 13.6% of total healthcare expenditure in 2010 [27]. Furthermore, patients have the option to use of health services that are not covered by insurance [21, 22, 27, 29].

Patients are also susceptible to income loss due to compensation for work incapacity being temporary, partial, or set at a subsistence level [22, 24]. For instance, job income is regularly compensated at about 70% by sickness benefit for 78 weeks or at about 50% by (early) retirement [22, 30]. Long-term unemployed individuals capable of working might collect a benefit of around 400 euros per month (unemployment benefit II) [24]. Moreover, income loss for many BC patients is likely; based on a study with German population-based data, only 64.3% of female BC survivors in active workforce at time of BC diagnosis returned to their former jobs, 19.4% reduced working hours within one year, and 5.4% left their job within 5 years after returning to work (RTW) [20].

Research on the financial burden of cancer patients has been increasing, particularly after 2010 [25, 31]. Four of every ten publications include US patients, while less than one of every ten includes patients located in Europe [12, 14, 25, 31–33]. For Germany, the literature is very limited [10, 22, 25]. Most publications, Apostolidis et al. [34], Mehlis et al. [30, 35, 36], Winkler et al. [37], and Witte et al. [38], were based on a study conducted among neuroendocrine tumor and colorectal cancer patients at the National Center for Tumor Diseases (NCT) Heidelberg. These studies showed the prevalence of OOP payments among cancer patients ranging between 78 and 87% [30, 34–38], and income loss was reported in 29 to 45% of cancer patients [30, 34–37]. In addition, based on a sample of German cancer patients, Büttner et al. [39] reported a monthly mean of 206 euros in OOP payments during the first 3 months after hospitalization. Between 3 and 15 months, monthly OOP expenses decreased to 179 euros, and after 15 months to 148 euros [39]. Similarly, the NCT studies suggested a monthly OOP payment that did not exceed 200 euros in 77% of affected patients, and a monthly income loss that exceeded 800 euros in 45% of patients reporting such losses [30, 34–36, 40]. Moreover, Hernandez et al. [26] estimated cancer patients to have lost between 26 and 28% of their job incomes within the first year after diagnosis, persisting 2 years thereafter.

The objective of this study is twofold: First, to quantify BC-related OOP payments and income loss among BC survivors in Germany who have lived for at least 5 years after diagnosis, and second, to explore socioeconomic and clinical factors of OOP payments and income loss.

## Methods

### Data and study population

The study population consists of participants of the CAESAR study, conducted by the German Cancer Research Center (DKFZ) in collaboration with six population-based cancer registries in Germany. Survivors from Bremen, Hamburg, North Rhine-Westphalia, Rhineland-Palatinate, Saarland, and Schleswig–Holstein were recruited from 2009 to 2011. To meet eligibility criteria, survivors were at least 5 years post breast, colorectal, or prostate cancer diagnosis confirmed between the years 1994 to 2004, and who were at the time 20 to 75 years of age. Further details regarding the methodology of the CAESAR study are available in several publications [20, 41–57].

In the present study, we analyzed survivors with a BC diagnosis (ICD-10: C50). We included BC survivors reporting either OOP or income loss, after following the selection criteria previously described in Doege, Thong [44]. The participants’ recruitment process as well as a description of the final sample are presented in the flow chart in Appendix 1.

Survivors were asked to report the OOP payments and income loss of the last 12 months prior to the completing of the questionnaire. Respondents had the option to provide the magnitude of OOP payments or income loss in euros or to endorse an unknown amount for each. OOP payments were asked independently for seven types of services where applicable, conformed by outpatient medical care, inpatient care, medicines and aids, other applications, alternative therapies, travel and transport, and domestic help/nursing staff. The first five OOP payment subcategories corresponded to direct medical costs, while the last two subcategories to direct non-medical costs. Work related loss of income was covered by one question in which respondents had to state the magnitude of such losses, constituting the indirect costs. In order to increase the quality of the data and to consider additional variables, CAESAR data were verified, supplemented, or replaced with data from cancer registries available within the CAESAR study. In addition, some of the variables in this study were constructed from information obtained from the Federal Statistical Office [58].

### Statistical analysis

Financial burden was evaluated by considering total OOP payments and total income loss separately. Total OOP payments were estimated as the sum of the seven OOP payment subcategories. If a value was missing for one of the seven OOP subcategories, but values were provided for at least one of the other OOP subcategories, then the missing value was assumed to be zero for that particular OOP subcategory. Or in other words, total OOP payments were estimated in this case as the sum of the values that were available. If values were missing for all of the seven OOP subcategories, then all missing values were assumed as such. Or in other words, total OOP payments were here set as missing. Total income loss was retrieved from the one survey question. Total OOP payments and total income loss ratios to household income were calculated as well. Given that the variable for monthly net household income was categorical, monthly household income was assumed to be the midpoint of the category range. For participants who selected “5000 euros or higher,” income was set to 6000 euros, as in previous studies [59–62]. Following the OECD modified equivalence scale, monthly household income was then adjusted by household size and composition and multiplied by 12 to obtain annual figures [63]. Both ratios were reported taking into account 0%, 1%, 2%, and 5% thresholds.

The relationship between financial burden and its risk factors were investigated using two-part models (TPMs). TPMs were chosen to address the large proportion of zeros in the data. For the first part of the TPMs, a logit model was employed to evaluate the probability of having any total OOP payments or total income loss. For the second part of the TPM, a generalized linear model (GLM) with a gamma distribution and a log link function was specified to estimate the intensity of total OOP payments and total income loss. A GLM tackles the presence of heteroscedasticity and distribution skewness of both dependent variables. Modified Park tests suggested a gamma distribution for the GLMs for total OOP payments and total income loss. The implementation of Box-Cox-tests for observations greater than zero supported the choice of a natural log transformation model for the link function in both GLMs. Following the general practice [64, 65], independent variables were included in both parts of the TPMs. All independent variables were included as categorical variables.

Two different TPMs were estimated, using first the total OOP payments and, second, the total income loss as the dependent variable. These are described by the following equation: *y*_*i*_|*x*_*i*_ = (*p*_*i*_|*x*_*i*_) × (*y*_*i*_|*y*_*i*_ > 0, *x*_*i*_), where *y* is the vector of the dependent variable, *x* the vector of independent variables, and *i* denotes for observation *i*; *p*_*i*_|*x*_*i*_ is the probability that *y*_*i*_ > 0 and *y*_*i*_|*y*_*i*_ > 0, *x*_*i*_ is the expected value of y_i_ conditional to *y*_*i*_ > 0. Independent variables were selected by considering previously published empirical evidence of potential risk factors related to OOP payments and income loss, comprising both socioeconomic and clinical individual characteristics. The final model specification included only independent variables showing a statistically significant bivariate association with the dependent variables (*p* ≤ 0.1). All risk factors incorporated in the final model specification are shown in Appendix 2, as well those that failed to be statistically significant in the bivariate analysis and were therefore excluded.

Predictive margins were calculated for the combined TPMs in order to identify overall risk factors. Based on this, the incremental effect was reported for each factor in the combined model, averaged over the included survivors, and supplemented by 95% confidence intervals (CI). Estimations were performed using robust standard errors and based on the delta method. The variance inflation factor (VIF) of all included independent variables suggested low probability for multicollinearity.

## Results

The final sample consists of 1344 BC survivors that provided information on OOP payments, and 905 on loss of income. The numbers and percentages for total OOP payments, OOP payments by subcategories, and total income loss within the last 12 months are presented in Table 1, and are reported as a proportion of the adjusted household income in Table 2. In addition, descriptive statistics for all independent variables included in the models are shown in Appendix 3. Total OOP payments were on average 294 euros among the 1344 survivors that reported OOP payments, and 566 euros (median 250 euros) among those that indicated OOP payments to be greater than zero. Overall, 646 survivors (48.1%) stated total OOP payments to be zero euro in the last 12 months. The aforementioned mean figures correspond to 2.2% and 4.3% of the adjusted annual household income respectively; this ratio is above 5% (1692 euros on average) for 10.3% of survivors. Moreover, OOP payment numbers by subcategory suggest that these were lowest for remedies and aids, averaging 14 euros, and highest for outpatient medical care, averaging 107 euros. When excluding zero observations, lowest OOP payments are found for remedies and aids, averaging 188 euros, and highest for domestic help and nursing staff, averaging 980 euros. For cumulative total OOP payments, the largest share was found for outpatient medical care at 36.4%. Direct medical costs were more prevalent but lower in payment amount than non-direct medical costs. Regarding income loss, it was on average 525 euros among the 905 that reported income loss, and 5463 euros (median 2500 euros) among those that indicated income loss to be greater than zero. This large difference responds to 818 survivors (90.4%) having reported income loss to be none in the last 12 months. Average figure corresponds to 1.8% and 18.8% of the adjusted annual baseline household income respectively; 6.3% of survivors had an income loss beyond the 5% ratio (7734 euros on average).

**Table 1:** OOP payments and income loss in last 12 months

OOP payments *N* = 1,344^1^/Income loss *N* = 905^1^	Whole sample		0 euro^2^	> 0 euro^2^			
	*N* (%)	Mean (SD)	N (%)	*N* (%)	Mean (SD)	Median (IQR)	Share in total amount (%)
Outpatient medical care	899 (66.9)	160 (423)	408 (30.4)	491 (36.5)	293 (538)	140 (240)	36.4
Inpatient medical care	837 (62.3)	37 (272)	745 (55.4)	92 (6.9)	336 (760)	200 (190)	7.8
Remedies and aids	888 (66.1)	22 (116)	786 (58.5)	102 (7.6)	188 (295)	90 (160)	4.9
Other applications	985 (73.3)	55 (166)	714 (53.1)	271 (20.2)	201 (266)	120 (140)	13.8
Alternative therapies	972 (72.3)	69 (283)	848 (63.1)	124 (9.2)	537 (615)	378 (515)	16.9
Travel and transport	861 (64.1)	22 (169)	771 (57.4)	90 (6.7)	215 (484)	100 (160)	4.9
Domestic help/nursing staff	1024 (76.2)	59 (297)	962 (71.6)	62 (4.6)	980 (751)	900 (880)	15.4
Total OOP payments^3^	1344 (100)	294 (755)	646 (48.1)	698 (51.9)	566 (972)	250 (550)	100.0
Total income loss	905 (100)	525 2804)	818 (90.4)	87 (9.6)	5463 (7440)	2500 (6560)	100.0

^1^Survivors with information in the past 12 months (0 or > 0)

^2^In the last 12 months

^3^At least for one OOP payment, subcategory information was provided

**Table 2:** OOP payments and income loss in previous 12 months as ratio of income

OOP payments/Income loss to adjusted household income^1^	OOP payments *N* = 1344			Income loss *N* = 905		
	*N* (%)	Mean (SD)	Mean to adjusted household income %^1^	*N* (%)	Mean (SD)	Mean to adjusted household income %^1^
0%	646 (48.1)	0 (0)	0.0	818 (90.4)	0 (0)	0.0
> 0%^2^	698 (51.9)	566 (972)	4.3	87 (9.6)	5462 (7441)	18.8
> 1%^2^	413 (30.7)	859 (1152)	6.7	82 (9.1)	5559 (7538)	19.2
> 2%^2^	289 (21.5)	1113 (1293)	9.0	72 (8.0)	6277 (7830)	21.7
> 5%^2^	138 (10.3)	1692 (1641)	15.4	57 (6.3)	7734 (8203)	26.5

^1^Adjusted annual household income according to the “OECD modified equivalence scale”

^2^When OOP payments, income loss and household income are provided

Results from the TPMs for total OOP payments and total income loss are presented in Tables 3 and 4, respectively. Both tables present outcomes for the logit and GLM models as well; however, we refer here to those from the combined models only. Coefficients are considered statistically significant if *p* ≤ 0.05. Compared to the age group 65 to 79 years, survivors in the age group 60 to 64 years had 196 euros less in total OOP payments, while those in the age group 50 and 59 years 187 euros less. Taking the group 9 years of education as baseline category, total OOP payments among survivors with 10 to 11 years of education were 95 euros higher. Relative to full-time work, unemployment was associated with a decrease of 143 euros in total OOP payments and (early) retirement with an increase of 140 euros. Diagnosis in a later cancer stage were linked to larger total OOP payments. When diagnosis at cancer stage I is the baseline category, survivors with a diagnosis in cancer stage II and in cancer stage III had more total OOP payments in amounts of 118 euros and 144 euros, respectively. Survivors with a diagnosis more than 10 years ago had 377 euros more in OOP payments compared to five to seven post diagnosis survivors. Compared to survivors without comorbidities, the OOP payments of survivors with at least one comorbidity was 139 euros higher. Having a hormone therapy was related to higher OOP payments in size of 96 euros, relative to not having it. The following variables were not found to be associated with total OOP payments in the combined model at statistically significant levels (*p* > 0.05): household income, disease management program (DMP) participation, psychosocial services usage, informational services usage, metastases occurrence, having a hospitalization, receiving chemotherapy, and receiving rehabilitation.

**Table 3:** Adjusted TPM for OOP payments and risk factors

OOP payments *N* = 1039^1^	Logistic^2^		GLM^3^		Combined	
Predictors	Coef. (*p*-value)	95% CI	Coef. (*p*-value)	95% CI	Margins (*p*-value)	95% CI
Constant	− 1.19* (0.01)	− 2.02 to − 0.37	5.87* (< 0.01)	5.18 to 6.56	298* (< 0.01)	260 to 336
Socioeconomic characteristics						
Age at diagnosis. Baseline: 65–79 years						
25 to 49 years	− 0.04 (0.88)	− 0.57 to 0.49	− 0.24 (0.28)	− 0.67 to 0.19	− 97 (0.32)	− 290 to 96
50 to 59 years	− 0.28 (0.21)	− 0.72 to 0.16	− 0.45* (0.02)	− 0.85 to -0.06	− 187* (0.03)	− 359 to − 15
60 to 64 years	− 0.21 (0.38)	− 0.67 to 0.25	− 0.52* (0.01)	− 0.93 to − 0.11	− 196* (0.03)	− 368 to − 25
Education. Baseline: ≤ 9 years						
10 to 11 years	0.12 (0.45)	− 0.19 to 0.44	0.29* (0.01)	0.07 to 0.50	94* (0.01)	25 to 164
≥ 12 years	− 0.11 (0.60)	− 0.50 to 0.29	0.44* (0.01)	0.11 to 0.78	117 (0.06)	− 3 to 236
Monthly adjusted household income^4^. Baseline: ≥ €1.500						
< €1.000	0.27 (0.16)	− 0.10 to 0.63	− 0.35* (0.02)	− 0.64 to − 0.07	− 68 (0.11)	− 151 to 15
€1.000 to < €1.500	0.39* (0.02)	0.06 to 0.72	− 0.13 (0.29)	− 0.36 to 0.11	6 (0.88)	− 76 to 89
Current employment. Baseline: full-time work						
Part-time work	0.50* (0.05)	0.01 to 0.98	− 0.05 (0.77)	− 0.40 to 0.30	29 (0.53)	− 62 to 119
Unemployed	− 0.42 (0.47)	− 1.55 to 0.71	− 0.85* (0.01)	− 1.46 to − 0.25	− 143* (< 0.01)	− 239 to − 47
Housewife	− 0.12 (0.66)	− 0.67 to 0.42	0.29 (0.17)	− 0.13 to 0.72	61 (0.31)	− 58 to 180
(Early-) retirement	0.02 (0.95)	− 0.48 to 0.51	0.48* (0.02)	0.09 to 0.87	140* (0.02)	24 to 256
Other/multiple	− 1.0 (0.05)	− 2.01 to 0.01	0.82* (0.03)	0.10 to 1.54	86 (0.54)	− 187 to 359
Clinical factors						
BC DMP participation. Baseline: no	0.51* (< 0.01)	0.19 to 0.84	− 0.08 (0.49)	− 0.31 to 0.15	33 (0.43)	− 49 to 115
Used psychosocial services. Baseline: no	0.18 (0.26)	− 0.14 to 0.51	0.19 (0.14)	− 0.06 to 0.43	80 (0.08)	− 10 to 171
Used informational services. Baseline: no	0.29* (0.05)	< 0.01 to 0.58	0.11 (0.31)	− 0.10 to 0.33	67 (0.06)	− 4 to 139
Cancer stage at BC diagnosis. Baseline: stage I						
Cancer stage II	0.29 (0.06)	− 0.01 to 0.59	0.30* (0.01)	0.08 to 0.52	118* (< 0.01)	43 to 193
Cancer stage III	0.67* (0.01)	0.17 to 1.18	0.24 (0.17)	− 0.10 to 0.57	144* (0.03)	14 to 273
Cancer stage IV	0.35 (0.52)	− 0.71 to 1.41	0.61 (0.07)	− 0.04 to 1.26	255 (0.17)	− 107 to 617
Time post BC diagnosis. Baseline: 5 to 7 years						
8 to 10 years	0.09 (0.57)	− 0.21 to 0.38	− 0.10 (0.41)	− 0.33 to 0.13	− 17 (0.63)	− 84 to 51
> 10 years	0.43 (0.10)	− 0.08 to 0.93	0.72* (< 0.01)	0.36 to 1.08	377* (< 0.01)	149 to 605
Comorbidities. Baseline: no	0.38* (0.02)	0.07 to 0.69	0.39* (< 0.01)	0.15 to 0.63	139* (< 0.01)	76 to 202
Metastases/recurrence/further cancer. Baseline: no	0.51* (0.02)	0.10 to 0.93	0.11 (0.42)	− 0.16 to 0.39	99 (0.08)	− 13 to 211
Hospitalization last 12 months. Baseline: no	0.51 (0.10)	− 0.09 to 1.11	0.18 (0.24)	− 0.12 to 0.48	126 (0.08)	− 17 to 270
Hormone therapy. Baseline: no	0.35* (0.01)	0.07 to 0.62	0.19 (0.06)	− 0.01 to 0.39	96* (0.01)	29 to 163
Chemotherapy. Baseline: no	0.25 (0.11)	− 0.06 to 0.57	0.05 (0.70)	− 0.21 to 0.31	45 (0.29)	− 37 to 127
Rehabilitation. Baseline: no	0.40* (0.01)	0.12 to 0.69	− 0.03 (0.80)	− 0.27 to 0.21	39 (0.31)	− 37 to 115
State fixed effects	Yes		Yes		Yes	

^1^1344 individuals with information for OOP payments (0 or > 0); 305 survivors excluded due to missing values in explanatory variables

^2^Wald chi^2^ (31) = 109.1. *p* < 0.01. Pseudo *R*^2^ = 0.09

^3^(1/df) deviance = 1.4. (1/df) Pearson = 1.5

^4^According to the “OECD modified equivalence scale”

^*^p ≤ 0.05

**Table 4:** Adjusted TPM for income loss and risk factors

Income loss *N* = 754^1^	Logistic^2^		GLM^3^		Combined	
Predictors	Coef. (*p*-value)	95% CI	Coef. (*p*-value)	95% CI	Margins (*p*-value)	95% CI
Constant	− 6.15* (< 0.01)	− 8.20 to − 4.10	5.73* (< 0.01)	3.88 to 7.58	762* (< 0.01)	331 to 1194
Socioeconomic characteristics						
Age at diagnosis. Baseline: 65–79 years						
25 to 49 years	1.68* (0.02)	0.31 to 3.04	1.29* (0.01)	0.32 to 2.26	1,137* (0.02)	220 to 2,054
50 to 59 years	1.41* (0.02)	0.20 to 2.62	1.98* (< 0.01)	1.06 to 2.91	1,939* (0.01)	415 to 3,462
60 to 64 years	− 0.29 (0.69)	− 1.71 to 1.14	− 1.01 (0.08)	− 2.13 to 0.12	− 63 (0.28)	− 179 to 52
Education. Baseline: ≤ 9 years						
10 to 11 years	0.36 (0.30)	− 0.33 to 1.04	− 0.27 (0.40)	− 0.88 to 0.35	− 3 (0.99)	− 604 to 598
≥ 12 years	− 0.01 (0.98)	− 0.91 to 0.89	0.1 (0.77)	− 0.59 to 0.79	74 (0.85)	− 694 to 842
Current employment. Baseline: full-time work						
Part-time work	0.89 (0.11)	− 0.20 to 1.98	1.08* (< 0.01)	0.56 to 1.61	608* (0.04)	33 to 1,184
Unemployed	1.04 (0.25)	− 0.72 to 2.81	0.28 (0.75)	− 1.45 to 2.01	247 (0.49)	− 452 to 946
Housewife	0.48 (0.47)	− 0.84 to 1.80	0.89 (0.16)	− 0.34 to 2.12	315 (0.27)	− 247 to 877
(Early) retirement	1.18* (0.03)	0.13 to 2.24	1.35* (0.03)	0.47 to 2.24	1066* (0.01)	243 to 1889
Other/multiple	2.38* (0.01)	0.60 to 4.16	0.34 (0.56)	− 0.80 to 1.47	821 (0.28)	− 675 to 2317
Current/last occupation. Baseline: non-manual employee						
Manual worker	− 0.24 (0.71)	− 1.51 to 1.02	− 0.29 (0.52)	− 1.17 to 0.59	− 146 (0.40)	− 489 to 196
Public official	0.92* (0.03)	0.07 to 1.76	0.61 (0.13)	− 0.18 to 1.41	910 (0.10)	− 164 to 1985
Self-employed	− 1.28 (0.11)	− 2.83 to 0.28	2.09* (0.04)	0.10 to 4.08	755 (0.58)	− 1939 to 3448
Other/multiple	− 0.05 (0.93)	− 1.23 to 1.12	2.6* (< 0.01)	1.86 to 3.35	4681 (0.13)	− 1444 to 10,806
Clinical factors						
BC DMP participation. Baseline: no	− 0.09 (0.79)	− 0.76 to 0.57	0.07 (0.78)	− 0.41 to 0.55	1 (1.00)	− 524 to 526
Used psychosocial services. Baseline: no	0.48 (0.13)	− 0.14 to 1.10	− 0.03 (0.91)	− 0.62 to 0.55	265 (0.45)	− 419 to 949
Used informational services. Baseline: no	0.15 (0.63)	− 0.46 to 0.76	− 0.48 (0.07)	− 1.01 to 0.05	− 298 (0.35)	− 925 to 329
Cancer stage at BC diagnosis. Baseline: stage I						
Cancer stage II	0.6 (0.06)	− 0.03 to 1.23	0.38 (0.12)	− 0.10 to 0.87	608* (0.05)	− 1 to 1,217
Cancer stage III	0.62 (0.24)	− 0.42 to 1.66	0.28 (0.48)	− 0.50 to 1.07	515 (0.33)	− 521 to 1552
Time post BC diagnosis. Baseline: 5 to 7 years						
8 to 10 years	− 0.96* (< 0.01)	− 1.57 to − 0.35	0.06 (0.88)	-0.68 to 0.79	-573 (0.21)	-1,471 to 324
> 10 years	− 0.62 (0.27)	− 1.73 to 0.49	− 1.02* (0.01)	− 1.82 to − 0.22	− 915* (0.04)	− 1,798 to − 31
Comorbidities. Baseline: no	1.98* (< 0.01)	1.01 to 2.95	< − 0.01 (0.99)	− 1.00 to 0.99	812* (< 0.01)	298 to 1325
Metastases/recurrence/further cancer. Baseline: no	0.89* (0.01)	0.19 to 1.60	0.59 (0.14)	− 0.20 to 1.38	1,504 (0.15)	− 550 to 3557
Chemotherapy. Baseline: no	0.16 (0.66)	− 0.56 to 0.89	0.82* (0.01)	0.24 to 1.39	550* (0.02)	89 to 1011
Rehabilitation. Baseline: no	0.42 (0.20)	− 0.22 to 1.05	0.08 (0.77)	− 0.44 to 0.59	296 (0.31)	− 276 to 868
State fixed effects	Yes		Yes		Yes	

^1^905 individuals with information for income loss (0 or > 0); 151 survivors excluded due to missing values for explanatory variables

^2^Wald chi^2^ (30) = 81.3. *p* < 0.01. Pseudo *R*^2^ = 0.23

^3^(1/df) deviance = 0.9. (1/df) Pearson = 0.6

^*^p ≤ 0.05

With respect to total income loss, results from the combined model suggests that, relative to survivors aged 65 to 79, those aged 25 to 49 and 50 to 59 had a much larger total income loss of 1137 euros and 1939 euros, respectively. Compared to full-time work, total income loss for part-time employees was 608 euros higher, and for survivors in (early) retirement 1066 euros higher. Survivors diagnosed in cancer stage II experienced a larger total income loss than those diagnosed in cancer stage I at the magnitude of 608 euros. With the 5 to 7 years post diagnosis group as baseline, those in the more than 10 years post diagnosis group had a significantly smaller total income loss in the size of 915 euros. Having at least one comorbidity was associated with a significantly larger total income loss of 812 euros, relative to not having any comorbidities. Survivors that received chemotherapy had significantly larger total income losses of 550 euros on average, compared to those that did not receive it. Total income loss was not predicted in the combined model at statistically significant levels (*p* > 0.05) by education level, occupation, DMP participation, usage of psychosocial services, usage of informational services, occurrence of metastases, and receiving rehabilitation.

### Sensitivity analysis

Generalized boxplots based on Tukey’s g-and-h were employed in order to estimate the impact of outliers in total OOP payments and total income loss [66–68]. Possible outliers were defined as any data point 1.5 interquartile below the first quartile and above the third quartile, and these were winsorized by replacing them with the highest amount observed after excluding outliers [66–68]. Coefficients in the combined model for total OOP payments remained similar in size and significance level. For total income loss, no potential outliers were observed. In addition, TPMs were re-estimated to include survivors for whom information from at least one independent variables was missing. In order to implement them, each missing independent variable was supplemented with a missing category. Coefficients for the combined models remained similar in size and significance levels for both total OOP payments and total income loss. These results are shown in Appendix 4 and Appendix 5.

## Discussion

The present study provides evidence to a topic that receives little attention in Germany. Because effective cancer treatments and medicines are commonly accessible and the extent of social security is ample, the financial burden of cancer for survivors is usually underestimated. Contrary to this view, results showed that 51.9% of long-term BC survivors experienced some form of OOP expenses averaging 566 euros in the last 12 months, which corresponded to 4.3% of their adjusted annual household income. The figure translates into 294 euros when considering as well survivors that do not incur in such expenses. Loss in income was less common, affecting only 9.6% of survivors; however, these losses were on average 5463 euros for the previous 12 months, or 18.8% of the adjusted annual household income. Average income losses were 525 euros when taking into account also survivors having reported losses to be none. Interestingly, income losses were also observed among survivors that are not actively working nor at a working age, suggesting that these recorded income losses do not necessarily relate to the main source of income.

The prevalence of OOP payments in this study is lower than those reported in previous research for Germany, which reported it to be between 78 and 87% [30, 34–38]. This difference might be explained, first, due to previous studies having considered cancer types different to BC. OOP payments are likely to differ by cancer type, and these have been observed to be higher for leukemia, lung and colorectal cancer than for BC [60, 62, 66–68]. And second, this study addressed long-term survivors. Previous literature suggests that long-term BC survivors are less affected by OOP payments than patients in the initial treatment phase due to the lower need for healthcare services [30, 36, 39, 40]. As for the extent of OOP payments, our results are similar to those of other studies that found OOP payments to be more common for direct medical costs (outpatient care in particular) and largest amounts to correspond to direct non-medical costs (domestic help and nursing staff) [39, 69, 70]. Furthermore, our results also suggest that OOP payments for long-term BC survivors in Germany rarely reach the catastrophic threshold (i.e., exceeding 15% of the annual household income) [71–73]. In the international context, OOP payments in long-term cancer survivors have been found to be higher in studies for other countries. For comparability purposes, here all figures have been transformed to 2011 euros. Baili et al. [74] estimated OOP payments at 160 euros per month on average in a sample of 5 to 10 years post diagnosis survivors in Italy. Likewise, Dean et al. [75] reported that BC survivors, with an average time of 12 years since diagnosis, incurred 1864 euros in OOP expenses. Country-specific regulations with respect to the type and extent of healthcare services covered by health insurances might drive these differences [12, 25, 76].

Income loss being less frequent but of a larger in amount than OOP payments is supported by previous empirical evidence [30, 35–37, 76]. For cancer survivors in Germany, the infrequency of income loss may be partially attributed to the existence of impatient cancer rehabilitation programs that support resumption to work [77]. In addition, employers, health insurers, unemployment, and pension insurances have financial incentives to support RTW [78]. Nevertheless, the prevalence of income loss in this study is lower in comparison to previous research for other cancer types, which estimated the occurrence of income loss to be between 28.7 and 44.8% [30, 34–37]. As with OOP payments, the type of cancer has been noted to influence the prevalence of income loss. For instance, Roelen et al. [79] found a shorter duration to full RTW in BC survivors relative to those with gastrointestinal, lung, or blood cancer [79]. Furthermore, substantial income losses are likely to respond to large proportion of (early) retirements in Germany [22, 24, 76]. Nevertheless, these were found in this study to be lower compared to those of cancer patients closer to the time of diagnosis [26, 30, 34–36, 40]. Our findings also suggest catastrophic healthcare costs as a result of income losses are rather uncommon in Germany. Studies in other countries have shown income losses to be more substantial. As stated above, we transformed other studies estimates into 2011 euros; comparability is, however, not straightforward given the different definitions and measurements of income loss. For Norway, Šaltytė Benth et al. [80] estimated annual income losses due to BC to be 3844 euros 5 years after diagnosis, 3489 euros 10 years after, and 2550 euros 13 years after. For the USA, Dean et al. [75] calculated productivity losses to be on average 1356 dollars per year for 12 year post diagnosis BC survivors, or 1474 euros. Chirikos et al. [81] reported an average reduction in annual household earnings of 3800 dollars, or 5170 euros, among women at least five years post BC diagnosis.

Furthermore, our study also found that predictors of reporting the highest amount of OOP expenses include age at time of diagnosis (65–79), education (10–11 years), (early) retirement, stage of diagnosis (stage III), time from diagnosis (more than 10 years), comorbidities (at least 1), and the use of rehabilitation services. Prior studies have also identified older and more educated BC patients to encounter higher OOP payments [17, 24, 59, 60, 67, 70, 74, 82–84]. Pisu et al. [85] and Newton, Johnson [68] also found OOP payments are the highest among survivors in (early) retirement, for whom a disability status is common and therefore make a higher use of healthcare services. Later cancer stages, longer time since diagnosis, and bearing comorbidities have often been reported as driving OOP payments [16, 67, 86, 87]. The category of stage IV cancer was not a predictor of higher OOP payments in our analysis, most likely due to the small number of observations for this group. The same might apply to survivors that underwent hospitalization, which usually report large OOP payments [60]. Having received chemotherapy is also a common determinant of OOP payments [67, 87]; however, our results do not show it to be statistically significant, presumably because its association with cancer stage, which is a predictor in our model.

Factors associated with income loss include age at time of diagnosis (50–59 years), (early) retirement, stage of diagnosis (stage II), time from diagnosis (5–7 years), comorbidities (at least 1) and receiving chemotherapy treatment. Being at a younger to middle age at diagnosis has been found in previous studies to be a predictive factor to income loss, as survivors of working age are more likely to be affected [24, 26, 36, 67, 76, 83, 86, 88, 89]. Other studies have also identified full time work to be negatively correlated with income loss [82, 89]. Nevertheless, occupation related income loss may be influenced by laws and regulations individual to each country [77, 78]. Longer time since diagnosis, diagnosis at earlier stages, bearing comorbidities, and receiving chemotherapy have been related with larger income losses in the literature as well [80, 82, 87, 88]. We found stage III cancer to be positively correlated with income loss, though not statistically significant, probably as a result of the low number of observations for the income loss regression.

### Strengths and limitations

Amongst the strengths of this analysis, we can highlight that unlike previous studies based on patient surveys, this analysis makes use of a large sample comprising survivors from different locations in Germany, delivering us a more accurate picture of the actual financial burden. In addition, the CAESAR study contains comprehensive information on socioeconomic and clinical characteristics of survivors, allowing us to test for a wide array of potential drivers of financial burden. And lastly, the model specification employed addresses the large proportion of zeros and skewedness in our sample, typical of healthcare expenditure data, providing a precise estimation.

A major limitation of this study is that we cannot distinguish between cases of non-utilization of healthcare services from those in which there was a full coverage or compensation. Both cases are marked in the survey with zero, and ideally, we would be able to differentiate them in order to understand the extent of OOP payments and income loss in the long run as a result of a BC diagnosis. We also assumed missing values for a single OOP subcategory to be zero when values were not missing for remaining OOP subcategories, which might underestimate the size of total OOP payments. Nevertheless, there is large a proportion of dropouts for the analysis due to missing values; therefore, conclusions are derived from a fraction of the study population. In addition, financial compensation by family and acquaintances are not addressed in our analysis and these might play an important role in financial burden alleviation beyond the coverage of health care and social security systems [59, 61, 90–92]. And finally, the study addresses financial burden at the long-term, providing a partial picture of the problematic. As previously mentioned, direct medical costs borne by patients are likely to be common during initial treatment and immediately after, nonetheless, we aim a highlighting that these are also substantial in the long-term and that those from direct non-medical costs and indirect costs can be at least as equally as important and persistent throughout a patients’ course of life.

## Conclusions

This study was the first to examine OOP payments and income loss attributable to BC among long-term survivors in Germany. Financial burden certain does not affect all BC survivors; in particular regarding income loss, however, in some cases, it can be substantial despite the provision of comprehensive healthcare and social security. For this reason, interventions designed to diminish OOP payments related to domestic help and nursing staff services are necessary, as well as for outpatient care. In addition, considering that income loss usually outweighs OOP payments, revising and expanding government programs to compensate income loss as well as supporting RTW should be prioritized. Furthermore, further research on the consequences of OOP payments and income loss is required, especially on health outcomes, in order to better understand long-term effects and patient needs.

## Figures and Tables

**Fig. 1:**
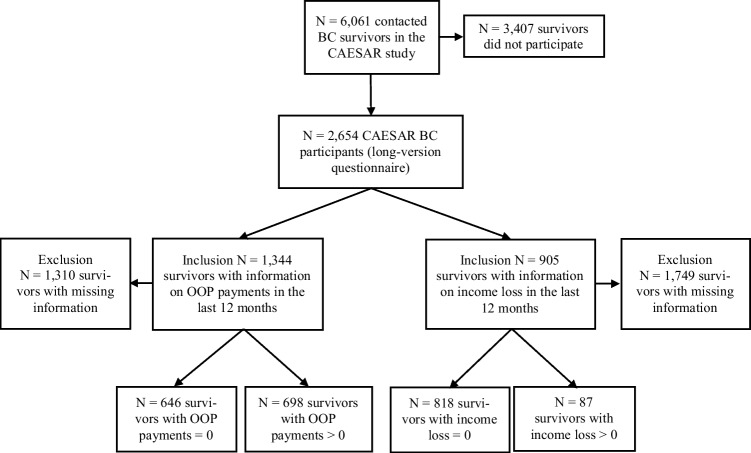
Recruitment process of CAESAR participants and final sample

## Data Availability

The datasets generated during and/or analyzed during the current study are available from the corresponding author on reasonable request.

## Appendix

**Table 5:** Study variables definitions, sources, and model inclusion

Variable	Definition	Source	TPM OOP payments	TPM income loss
Gender	Dummy variable coded 1 if the individual is female at the time of the survey, and 0 otherwise	Registry	Excluded	Excluded
Age at diagnosis 25 to 49 years	Dummy variable coded 1 if the individual is aged 25 to 49 years at diagnosis, and 0 otherwise	Registry	Included	Included
Age at diagnosis 50 to 59 years	Dummy variable coded 1 if the individual is aged 50 to 59 years at diagnosis, and 0 otherwise	Registry	Included	Included
Age at diagnosis 60 to 64 years	Dummy variable coded 1 if the individual is aged 60 to 64 years at diagnosis, and 0 otherwise	Registry	Included	Included
Age at diagnosis 65 to 79 years	Baseline category. Dummy variable coded 1 if the individual is aged 65 to 79 years at diagnosis, and 0 otherwise	Registry	Included	Included
Nationality	Dummy variable coded 1 if the individual has no German nationality at the time of the survey, and 0 otherwise	CAESAR study	Excluded	Excluded
Education of ≤ 9 years	Baseline category. Dummy variable coded 1 if the individual is educated 9 years or less at the time of the survey, and 0 otherwise	CAESAR study	Included	Included
Education of 10 to 11 years	Dummy variable coded 1 if the individual is educated 10 to 11 years at the time of the survey, and 0 otherwise	CAESAR study	Included	Included
Education of ≥ 12 years	Dummy variable coded 1 if the individual is educated at least 12 years at the time of the survey, and 0 otherwise	CAESAR study	Included	Included
Married /with partner	Dummy variable coded 1 if the individual is not married/with a partner at the time of the survey, and 0 otherwise	CAESAR study	Excluded	Excluded
Densely population density	Baseline category. Dummy variable coded 1 if the place of residence of the individual has a densely population density at the time of the survey, and 0 otherwise	Registry, Destatis	Excluded	Excluded
Medium population density	Dummy variable coded 1 if the place of residence of the individual has a medium population density at the time of the survey, and 0 otherwise	Registry, Destatis	Excluded	Excluded
Sparsely population density	Dummy variable coded 1 if the place of residence of the individual has a sparsely population density at the time of the survey, and 0 otherwise	Registry, Destatis	Excluded	Excluded
Monthly adjusted household income < €1000	Dummy variable coded 1 if the individual has an adjusted monthly household income of < €1000 according to the “OECD modified equivalence scale” at the time of the survey, and 0 otherwise. Based on monthly net household income	CAESAR study	Included	Excluded
Monthly adjusted household income €1000 to < €1500	Dummy variable coded 1 if the individual has an adjusted monthly household income of €1000 to < €1500 according to the “OECD modified equivalence scale” at the time of the survey, and 0 otherwise. Based on net monthly net household income	CAESAR study	Included	Excluded
Monthly adjusted household income ≥ €1500	Baseline category. Dummy variable coded 1 if the individual has an adjusted monthly household income of ≥ €1500 according to the “OECD modified equivalence scale” at the time of the survey, and 0 otherwise. Based on net monthly net household income	CAESAR study	Included	Excluded
Full-time work	Baseline category. Dummy variable coded 1 if the individual is working full-time at the time of the survey, and 0 otherwise	CAESAR study	Included	Included
Part-time work	Dummy variable coded 1 if the individual is working part-time at the time of the survey, and 0 otherwise	CAESAR study	Included	Included
Unemployed	Dummy variable coded 1 if the individual is unemployed at the time of the survey, and 0 otherwise	CAESAR study	Included	Included
Housewife	Dummy variable coded 1 if the individual is a housewife at the time of the survey, and 0 otherwise	CAESAR study	Included	Included
(Early) retirement	Dummy variable coded 1 if the individual is in (early) retirement at the time of the survey, and 0 otherwise	CAESAR study	Included	Included
Other/multiple employments	Dummy variable coded 1 if the individual has other or multiple employments at the time of the survey, and 0 otherwise	CAESAR study	Included	Included
Non-manual employee	Baseline category. Dummy variable coded 1 if the individual is/was at last a non-manual employee at the time of the survey, and 0 otherwise	CAESAR study	Excluded	Included
Manual worker	Dummy variable coded 1 if the individual is/was at last a manual worker at the time of the survey, and 0 otherwise	CAESAR study	Excluded	Included
Public official	Dummy variable coded 1 if the individual is/was at last a public official at the time of the survey, and 0 otherwise	CAESAR study	Excluded	Included
Self-employed	Dummy variable coded 1 if the individual is/was at last self-employed at the time of the survey, and 0 otherwise	CAESAR study	Excluded	Included
Other/multiple occupations	Dummy variable coded 1 if the individual has/had at last other or multiple occupations at the time of the survey, and 0 otherwise	CAESAR study	Excluded	Included
BC DMP participation	Dummy variable coded 1 if the individual has/had a participation in a BC DMP at the time of the survey, and 0 otherwise	CAESAR study	Included	Included
Used psychosocial services	Dummy variable coded 1 if the individual has used at least one psychosocial service since BC diagnosis at the time of the survey, and 0 otherwise. Includes psycho-oncologists in a hospital/community-based service centre and patient support groups	CAESAR study	Included	Included
Used informational services	Dummy variable coded 1 if the individual has used at least one informational service since BC diagnosis at the time of the survey, and 0 otherwise. Includes information via telephone, internet, brochures	CAESAR study	Included	Included
Cancer stage I at BC diagnosis	Baseline category. Dummy variable coded 1 if the individual has cancer stage I at first diagnosis according to the TNM classification, and 0 otherwise	CEASAR study and registry	Included	Included
Cancer stage II at BC diagnosis	Dummy variable coded 1 if the individual has cancer stage II at first diagnosis according to the TNM classification, and 0 otherwise	CEASAR study and registry	Included	Included
Cancer stage III at BC diagnosis	Dummy variable coded 1 if the individual has cancer stage III at first diagnosis according to the TNM classification, and 0 otherwise	CEASAR study and registry	Included	Included
Cancer stage IV at BC diagnosis	Dummy variable coded 1 if the individual has cancer stage IV at first diagnosis according to the TNM classification, and 0 otherwise	CEASAR study and registry	Included	Included
5 to 7 years post BC diagnosis	Baseline category. Dummy variable coded 1 if the individual was diagnosed with BC five to seven years ago at the time of the survey, and 0 otherwise	CAESAR study	Included	Included
8 to 10 years post BC diagnosis	Dummy variable coded 1 if the individual was diagnosed with BC eight to ten years ago at the time of the survey, and 0 otherwise	CAESAR study	Included	Included
> 10 years post BC diagnosis	Dummy variable coded 1 if the individual was diagnosed with BC > 10 years ago at the time of the survey, and 0 otherwise	CAESAR study	Included	Included
Comorbidity/ies	Dummy variable coded 1 if the individual has/had at least one comorbidity at the time of the survey, and 0 otherwise	CAESAR study	Included	Included
Metastases/recurrence/further cancer	Dummy variable coded 1 if the individual have/had metastases/recurrence/a further cancer diagnosis since their BC diagnosis, and 0 otherwise	CAESAR study	Included	Included
Hospitalization within the last 12 months	Dummy variable coded 1 if the individual had a hospitalization within the last 12 months at the time of the survey, and 0 otherwise	CAESAR study	Included	Excluded
Surgery	Dummy variable coded 1 if the individual received surgery since BC diagnosis, and 0 otherwise. Included are breast removal mastectomy, conservation, reconstruction and ablation	CAESAR study	Excluded	Excluded
Hormone therapy	Dummy variable coded 1 if the individual received hormone therapy since BC diagnosis, and 0 otherwise	CAESAR study	Included	Excluded
Radiotherapy	Dummy variable coded 1 if the individual received radiotherapy since BC diagnosis, and 0 otherwise	CAESAR study	Excluded	Excluded
Chemotherapy	Dummy variable coded 1 if the individual received chemotherapy since BC diagnosis, and 0 otherwise	CAESAR study	Included	Included
Axillary lymph node dissection	Dummy variable coded 1 if the individual received axillary lymph node dissection since BC diagnosis, and 0 otherwise	CAESAR study	Excluded	Excluded
Rehabilitation	Dummy variable coded 1 if the individual received rehabilitation since BC diagnosis, and 0 otherwise	CAESAR study	Included	Included

**Table 6:** Descriptive statistics for study population

Socioeconomic and clinical factors	Study population *N* = 2654	OOP payments *N* = 1344^1^	Income loss *N* = 905^1^
	*n*/value (%)	*n*/value (%)	*n*/value (%)
Female	2654 (100.0)	1344 (100.0)	905 (100.0)
Age at diagnosis			
25 to 49 years	632 (23.8)	417 (31.0)	266 (29.4)
50 to 59 years	821 (30.9)	440 (32.7)	304 (33.6)
60 to 64 years	598 (22.5)	278 (20.7)	198 (21.9)
65 to 79 years	598 (22.5)	208 (15.5)	137 (15.1)
Missing	5 (0.2)	1 (0.1)	0 (0.0)
Nationality			
German	2,459 (92.7)	1,249 (92.9)	842 (93.0)
Not German	42 (1.6)	24 (1.8)	16 (1.8)
Missing	153 (5.8)	71 (5.3)	47 (5.2)
Education			
≤ 9 years	1,355 (51.1)	551 (41.0)	348 (38.5)
10 to 11 years	871 (32.8)	520 (38.7)	352 (38.9)
≥ 12 years	389 (14.7)	263 (19.6)	196 (21.7)
Missing	39 (1.5)	10 (0.7)	9 (1.0)
Married/with partner			
Yes	1939 (73.1)	1019 (75.8)	686 (75.8)
No	714 (26.9)	325 (24.2)	219 (24.2)
Missing	1 (< 0.1)	0 (0.0)	0 (0.0)
Urbanization of place of residence			
Densely population density	775 (29.2)	405 (30.1)	278 (30.7)
Medium population density	991 (37.3)	508 (37.8)	338 (37.4)
Sparsely population density	324 (12.2)	157 (11.7)	111 (12.3)
Missing	564 (21.3)	274 (20.4)	178 (19.7)
Monthly adjusted household income^2^			
< €1.000	650 (24.5)	262 (19.5)	147 (16.2)
€1.000 to < €1.500	640 (24.1)	319 (23.7)	201 (22.2)
≥ €1.500	1,130 (42.6)	689 (51.3)	509 (56.2)
Missing	234 (8.8)	74 (5.5)	48 (5.3)
Current employment			
Full-time work	208 (7.8)	150 (11.2)	109 (12.0)
Part-time work	406 (15.3)	259 (19.3)	173 (19.1)
Unemployed	35 (1.3)	21 (1.6)	11 (1.2)
Housewife	542 (20.4)	222 (16.5)	135 (14.9)
(Early) retirement	1336 (50.3)	650 (48.4)	450 (49.7)
Other/multiple	76 (2.9)	35 (2.6)	21 (2.3)
Missing	51 (1.9)	7 (0.5)	6 (0.7)
Current/last occupation			
Non-manual employee	1512 (57.0)	858 (63.8)	572 (63.2)
Manual worker	315 (11.9)	109 (8.1)	70 (7.7)
Public official	144 (5.4)	98 (7.3)	87 (9.6)
Self-employed	171 (6.4)	91 (6.8)	60 (6.6)
Other/multiple	373 (14.1)	148 (11.0)	92 (10.2)
Missing	139 (5.2)	40 (3.0)	24 (2.7)
Breast cancer DMP participation since BC diagnosis			
Yes	468 (17.6)	290 (21.6)	182 (20.1)
No	2056 (77.5)	1008 (75.0)	694 (76.7)
Missing	130 (4.9)	46 (3.4)	29 (3.2)
Used psychosocial services since BC diagnosis			
Yes	522 (19.7)	341 (25.4)	203 (22.4)
No	2086 (78.6)	992 (73.8)	695 (76.8)
Missing	46 (1.7)	11 (0.8)	7 (0.8)
Used informational services since BC diagnosis			
Yes	1211 (45.6)	729 (54.2)	475 (52.5)
No	1389 (52.3)	605 (45.0)	425 (47.0)
Missing	54 (2.0)	10 (0.7)	5 (0.6)
Cancer stage (TNM classification) at BC diagnosis			
Stage I	1121 (42.2)	569 (42.3)	410 (45.3)
Stage II	1094 (41.2)	548 (40.8)	354 (39.1)
Stage III	203 (7.7)	114 (8.5)	69 (7.6)
Stage IV	31 (1.2)	17 (1.3)	8 (0.9)
Missing	205 (7.7)	96 (7.1)	64 (7.1)
Time post BC diagnosis			
5 to 7 years	1152 (43.4)	589 (43.8)	409 (45.2)
8 to 10 years	1172 (44.2)	591 (44.0)	385 (42.5)
> 10 years	325 (12.3)	163 (12.1)	111 (12.3)
Missing	5 (0.2)	1 (0.1)	0 (0.0)
Current/past comorbidities			
Yes	1970 (74.2)	982 (73.1)	623 (68.8)
No	675 (25.4)	362 (26.9)	282 (31.2)
Missing	9 (0.3)	0 (0.0)	0 (0.0)
Metastases/recurrence/further cancer since BC diagnosis			
Yes	363 (13.7)	199 (14.8)	115 (12.7)
No	2272 (85.6)	1136 (84.5)	783 (86.5)
Missing	19 (0.7)	9 (0.7)	7 (0.8)
Hospitalization last 12 months			
Yes	167 (6.3)	91 (6.8)	55 (6.1)
No	2414 (90.7)	1232 (91.7)	837 (92.5)
Missing	73 (2.75)	21 (1.6)	13 (1.4)
Received surgery			
Yes	2477 (93.3)	1282 (95.4)	859 (94.9)
No	150 (5.65)	57 (4.2)	42 (4.6)
Missing	27 (1.02)	5 (0.4)	4 (0.4)
Received hormone therapy			
Yes	1194 (45.0)	692 (51.5)	459 (50.7)
No	1240 (46.7)	586 (43.6)	401 (44.3)
Missing	220 (8.3)	66 (4.9)	45 (5.0)
Received radiotherapy			
Yes	2188 (82.4)	1120 (83.3)	756 (83.5)
No	416 (15.7)	209 (15.6)	139 (15.4)
Missing	50 (1.9)	15 (1.1)	10 (1.1)
Received chemotherapy			
Yes	1553 (58.5)	806 (60.0)	521 (57.6)
No	997 (37.6)	506 (37.7)	367 (40.6)
Missing	104 (3.9)	32 (2.4)	17 (1.9)
Received axillary lymph node dissection			
Yes	2447 (92.2)	1268 (94.4)	850 (93.9)
No	150 (5.65)	60 (4.5)	44 (4.9)
Missing	57 (2.15)	16 (1.2)	11 (1.2)
Received rehabilitation			
Yes	1571 (59.2)	856 (63.7)	537 (59.3)
No	975 (36.7)	459 (34.2)	351 (38.8)
Missing	108 (4.07)	29 (2.2)	17 (1.9)

^1^Survivors with information in the past 12 months (0 or > 0)

^2^According to the “OECD modified equivalence scale”

**Table 7:** Adjusted TPM for OOP payments and risk factors with missing categories

OOP payments *N* = 1343^1^	Combined model^2^	
Predictors	Margins (*p*-value)	95% CI
Constant	297* (< 0.01)	260 to 335
Sociodemographic characteristics		
Age at diagnosis. Baseline: 65–79 years		
25 to 49 years	− 23 (0.78)	− 179 to 134
50 to 59 years	− 128 (0.06)	− 261 to 5
60 to 64 years	− 149* (0.03)	− 281 to − 16
Missing	− 320* (< 0.01)	− 475 to − 164
Education. Baseline: ≤ 9 years		
10 to 11 years	100* (< 0.01)	35 to 164
≥ 12 years	138* (0.02)	27 to 250
Missing	− 145* (0.01)	− 249 to − 40
Monthly adjusted household income^3^. Baseline: ≥ €1.500		
< €1.000	− 75* (0.05)	− 151 to 1
€1.000 to < €1.500	− 9 (0.81)	− 86 to 67
Missing	5 (0.96)	− 181 to 190
Current employment. Baseline: full-time work		
Part-time work	30 (0.45)	− 48 to 107
Unemployed	− 102* (0.04)	− 200 to − 5
Housewife	107 (0.06)	− 5 to 219
(Early-) retirement	134* (0.01)	34 to 234
Other/multiple	123 (0.28)	− 99 to 345
Missing	633 (0.30)	− 564 to 1830
Clinical factors		
BC DMP participation. Baseline: no		
Yes	50 (0.22)	− 30 to 131
Missing	19 (0.88)	− 227 to 265
Used psychosocial services. Baseline: no		
Yes	79 (0.06)	− 3 to 161
Missing	413 (0.20)	− 224 to 1049
Used informational services. Baseline: no		
Yes	53 (0.12)	− 13 to 120
Missing	− 137 (0.12)	− 309 to 34
Cancer stage at BC diagnosis. Baseline: stage I		
Cancer stage II	138* (< 0.01)	67 to 209
Cancer stage III	150* (0.02)	22 to 277
Cancer stage IV	210 (0.20)	− 111 to 531
Missing	37 (0.49)	− 68 to 142
Time post BC diagnosis. Baseline: 5 to 7 years		
8 to 10 years	− 17 (0.60)	− 80 to 46
> 10 years	297* (< 0.01)	104 to 489
Missing	-	-
Comorbidities. Baseline: no		
Yes	174* (< 0.01)	118 to 230
Missing	-	-
Metastases/recurrence/further cancer. Baseline: no		
Yes	100* (< 0.01)	7 to 194
Missing	1632 (0.23)	− 1002 to 4267
Hospitalization last 12 months. Baseline: no		
Yes	121 (0.08)	− 12 to 255
Missing	− 131* (0.03)	− 246 to − 16
Hormone therapy. Baseline: no		
Yes	99* (< 0.01)	37 to 162
Missing	180 (0.08)	− 24 to 384
Chemotherapy. Baseline: no		
Yes	33 (0.38)	− 41 to 107
Missing	76 (0.60)	− 205 to 356
Rehabilitation. Baseline: no		
Yes	50 (0.15)	− 19 to 119
Missing	− 77 (0.27)	− 216 to 61
State fixed effects	Yes	

^1^1344 individuals with information for OOP payments (0 or > 0)

^2^Wald chi^2^ (43) = 143.1. *p* < 0.01. Pseudo *R*^2^ = 0.09. (1/df) deviance = 1.4. (1/df) Pearson = 1.5

^3^According to the “OECD modified equivalence scale”

^*^*p* ≤ 0.05

**Table 8:** Adjusted TPM for income loss and risk factors with missing categories

Income loss *N* = 861^1^	Combined model^2^	
Predictors	Margins (*p*-value)	95% CI
Constant	822* (0.01)	166 to 1478
Sociodemographic characteristics		
Age at diagnosis. Baseline: 65–79 years		
25 to 49 years	1240* (0.02)	211 to 2,270
50 to 59 years	1850* (0.03)	224 to 3477
60 to 64 years	− 44 (0.39)	− 144 to 56
Missing	-	-
Education. Baseline: ≤ 9 years		
10 to 11 years	176 (0.64)	− 568 to 920
≥ 12 years	17 (0.97)	− 826 to 861
Missing	− 512 (0.17)	− 1237 to 213
Current employment. Baseline: full-time work		
Part-time work	632* (0.04)	38 to 1227
Unemployed	61 (0.72)	− 269 to 392
Housewife	321 (0.27)	− 247 to 889
(Early-) retirement	951* (0.01)	228 to 1674
Other/multiple	2044 (0.35)	− 2205 to 6,293
Missing	4146 (0.34)	− 4146 to 12,693
Current/last occupation. Baseline: non-manual employee		
Manual worker	− 98 (0.59)	− 452 to 255
Public official	938 (0.15)	− 326 to 2201
Self-employed	1327 (0.58)	− 3365 to 6019
Other/multiple	4573 (0.11)	− 1035 to 10,180
Missing	107 (0.76)	− 585 to 799
Clinical factors		
BC DMP participation. Baseline: no		
Yes	206 (0.51)	− 403 to 815
Missing	-	-
Used psychosocial services. Baseline: no		
Yes	506 (0.19)	− 253 to 1266
Missing	-	-
Used informational services. Baseline: no		
Yes	− 486 (0.30)	− 1396 to 423
Missing	-	-
Cancer stage at BC diagnosis. Baseline: stage I		
Cancer stage II	687 (0.06)	− 687 to − 39
Cancer stage III	530 (0.35)	530 to -583
Missing	1033 (0.34)	− 1083 to 3149
Time post BC diagnosis. Baseline: 5 to 7 years		
8 to 10 years	− 748 (0.20)	− 748 to − 1898
> 10 years	− 938 (0.09)	− 2032 to 156
Missing	-	-
Comorbidities. Baseline: no		
Yes	1033* (0.03)	117 to 1948
Missing	-	-
Metastases/recurrence/further cancer. Baseline: no		
Yes	1108 (0.12)	− 273 to 2489
Missing	1254 (0.65)	− 4139 to 6647
Chemotherapy. Baseline: no		
Yes	516 (0.12)	516 to − 130
Missing	− 445* (0.03)	− 445 to − 842
Rehabilitation. Baseline: no		
Yes	166 (0.64)	− 518 to 850
Missing	− 751* (0.03)	− 751 to − 1417
State fixed effects	Yes	

^1^861 individuals with information for OOP payments (0 or > 0)

^2^Wald chi^2^ (37) = 108.0. *p* < 0.01. Pseudo *R*^2^ = 0.26. (1/df) Deviance = 1.0. (1/df) Pearson = 0.7

^*^*p* ≤ 0.05
